# A Compact Energy Harvesting System for Outdoor Wireless Sensor Nodes Based on a Low-Cost In Situ Photovoltaic Panel Characterization-Modelling Unit

**DOI:** 10.3390/s17081794

**Published:** 2017-08-04

**Authors:** Diego Antolín, Nicolás Medrano, Belén Calvo, Pedro A. Martínez

**Affiliations:** Group of Electronic Design (GDE), Aragón Institute for Engineering Research (I3A), Departamento de Ingeniería Electrónica y Comunicaciones, Universidad de Zaragoza, C/ Pedro Cerbuna 12, Zaragoza 50009, Spain; dantolin@unizar.es (D.A.); becalvo@unizar.es (B.C.); pemar2@unizar.es (P.A.M.)

**Keywords:** maximum power point tracking, solar panel, artificial neural network, PV modelling, energy harvesting, wireless sensor network, cyber-physical systems

## Abstract

This paper presents a low-cost high-efficiency solar energy harvesting system to power outdoor wireless sensor nodes. It is based on a Voltage Open Circuit (VOC) algorithm that estimates the open-circuit voltage by means of a multilayer perceptron neural network model trained using local experimental characterization data, which are acquired through a novel low cost characterization system incorporated into the deployed node. Both units—characterization and modelling—are controlled by the same low-cost microcontroller, providing a complete solution which can be understood as a virtual pilot cell, with identical characteristics to those of the specific small solar cell installed on the sensor node, that besides allows an easy adaptation to changes in the actual environmental conditions, panel aging, etc. Experimental comparison to a classical pilot panel based VOC algorithm show better efficiency under the same tested conditions.

## 1. Introduction

**Figure 1:**
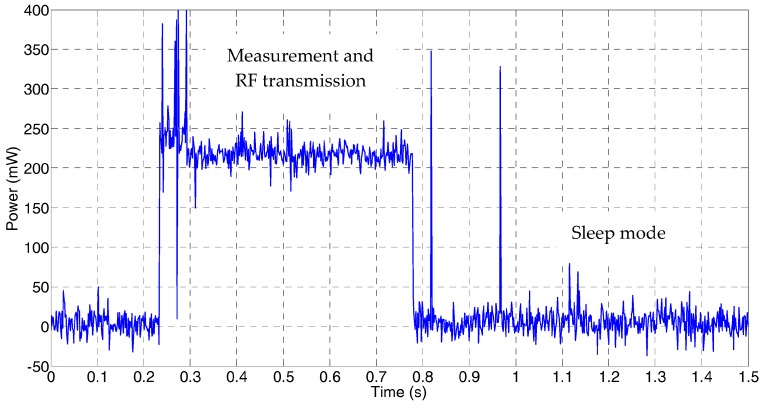
Typical power consumption profile for an environmental wireless sensor node.

Energy harvesting (EH) is a main research field in cyber-physical systems reliability, especially in portable and unattended environmental sensing applications, such as wireless sensor networks (WSN) deployed on extensive outdoor areas monitoring ambient conditions as temperature, light, pressure, and humidity or air pollutants. Figure 1 shows a typical power consumption profile for a sensor node within a WSN intended for environmental monitoring. Most of the time the nodes are set to a low power state (sleep mode in Figure 1), being periodically wake up to acquire the desired environmental parameters, which are then conveyed to the network host. The slow variation of environmental conditions allows programming sleep times of tens of minutes, while the power-hungry measurement and radio frequency (RF) transmission processes take a few seconds or less. Depending on the network configuration, some of these wireless nodes can be configured to receive data from the neighbouring devices, thus extending the surveyed area using intermediate routers for data transmission in a multi-hop architecture, with an additional cost in energy due to the RF transmission. According to the current energy available in the node, a suitable power management requires the use of an optimized cross-layer framework [1] for a precise adjustment of several main parameters in the sensor node as duty cycle, RF power transmission, node data routing and energy harvesting from the environment. Actually, a suitable node sensor power management together with an efficient energy harvesting technique is critical to increase the sensor network autonomy while reducing the required maintenance operations.

In these WSN monitoring applications in natural areas, reviewing the different techniques to harvest energy from the environment, solar energy is the preferential choice because of its availability and predictability [2,3,4,5,6]. The solar power is converted to electric energy through low-cost and low-size photovoltaic (PV) panels. Thus, to successfully design an efficient EH system, one first key issue is having an accurate PV model to reliably predict the panel behaviour under the expected working conditions, with the objective of optimizing the energy transfer from the panel to the energy storage device, by selecting the suitable electric load connected to the panel as a function of the maximum generated power, which in turn depends on the physical solar cell characteristics and ambient working conditions.

The behavioural description of large-size high-power PV panels and PV arrays is typically based on simulation/modelling algorithms using Matlab and/or Simulink [7,8,9,10], specific high level mathematical simulation models [11,12], or electrical–based models [13] that extract the required parameters from the manufacturer datasheet, from experimental PV characterization data (this one being the optimum choice) or from a combination of both. A PV panel characterization system must measure the power-to-voltage characteristic under different irradiance and temperature values, at different working conditions obtained by changing the electric load connected to the panel. Thus, a complete PV panel characterization station requires the use of bulky and expensive instruments (solar simulators, pyranometers, programmable power loads), so that these infrastructures are only worthy for large power photovoltaic panels.

In the case of low-cost small PV cells, such as those usually used in wireless sensor nodes, models provided by manufacturers are limited to an approximate behaviour without an accurate description, thus jeopardizing the related efficiency in the power management and transmission system. To overcome this limitation, this work first introduces a low-cost and compact PV panel characterization system, designed to be included as a part of the sensor node. The acquired experimental data are then used to complete a PV panel modelling based on the use of artificial neural networks (ANN). With this approach, it is possible to characterize in situ the photovoltaic cell, and re-characterizing it periodically, upgrading the panel model according to the actual working parameters, and including effects of efficiency lost due to aging, solar radiation variations related to seasonality or dust deposition over the active area. The final goal is the implementation of a maximum power point tracking system based on a Voltage Open Circuit (VOC) algorithm that models the solar panel to reproduce its open-circuit behaviour under the real environmental working conditions in order to speed up the load of an energy storage system in low form factor wireless sensor nodes, maintaining as objectives maximum efficiency, low cost and minimization of visual impact.

The paper is organized as follows: Section 2 presents the compact and portable PV panel characterization system designed for locally accomplish parameter extraction in low-cost solar panels, utilizing a small form-factor PV device that powers a wireless sensor node. Section 3 addresses the modelling of the photovoltaic panel, with a multilayer perceptron neural network as fitting tool using the data collected from the previous characterization system. Section 4 presents the energy harvesting system for the sensor node mounting the panel, based on a DC-DC boost converter with a maximum power point tracking VOC algorithm that relies on the developed PV modelling approach to track the open circuit voltage. Experimental results confirm the achievement of better efficiency compared to a classical VOC technique based on the use of a pilot panel, thus allowing faster charge of the energy storage device.

## 2. In Situ Photovoltaic Panel Characterization

The proposed PV panel characterization system follows the classical approach, adapted for low-cost solar panels and it owns the characteristic of portability: it allows one to characterize the PV cell in the same site where the device is deployed, thus providing a local accurate behavior model that considers the real external parameters affecting the PV panel operation (light, temperature), as well as the real voltage and current provided by the panel at different programmable loads.

**Figure 2:**
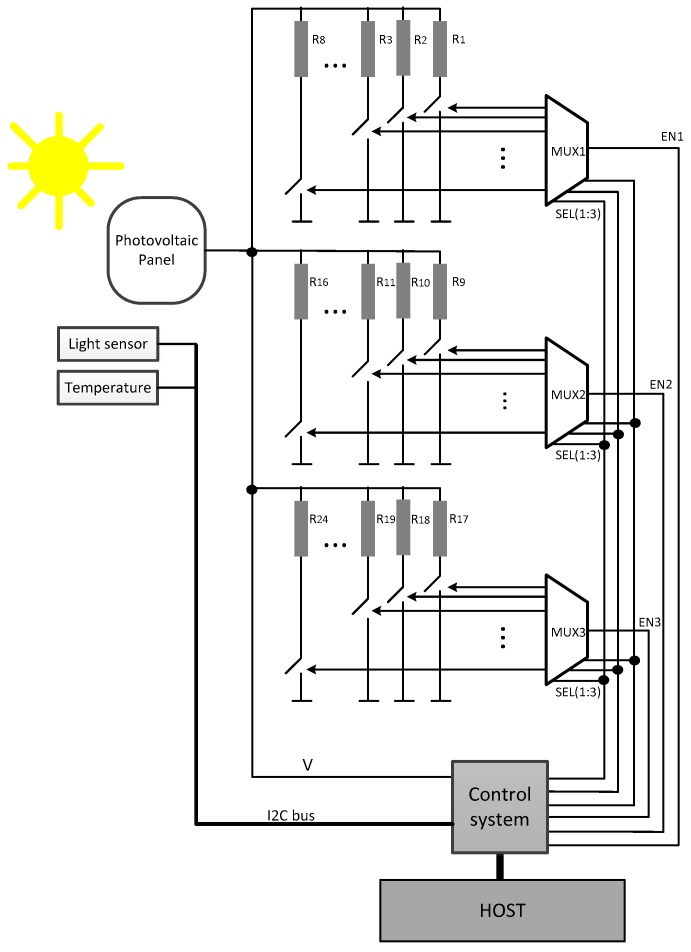
Proposed PV panel characterization system.

It consists of three main elements (Figure 2): a compact variable load connected to the device under test; a control and acquisition system that selects the load value connected to the PV cell at each moment and reads the data provided by the PV cell at actual temperature and light conditions; and the system software, which coordinates the full system operation. The PV panel selected is a MC-SP0.8-NF-GCS from Multicomp (Premier Farnell, Leeds, UK); its main parameters are: 800 mW maximum power, 4.8 V open circuit voltage and a temperature operation range from −40 °C to +85 °C.

### 2.1. Variable Load Module

The custom variable load consists on a set of 24 fixed value resistors (Table 1), which can be selected by means of three 1:8 demultiplexers that enable or disable a set of ultra-low on-resistance MOSFET transistor switches in series with the loads, connecting the PV panel to each selected resistor at a time (Figure 2). The selection of resistor values attends to the PV panel behaviour, covering from near open circuit for high resistors, to short circuit for the lowest resistor value.

Their overall real values have been measured using a Keysight (Santa Rosa, CA, USA) 34410A 6 ½ digital multimeter (DMM) using four-wire technique. In this way, the accurate knowledge of the resistor values allows estimate the value of the power transferred to the load at a certain voltage without requiring the current measurement, simplifying the data acquisition. The operation of this module is controlled by a low-cost microcontroller that selects the panel load and reads the measurements, locally storing the values before being sent to a host system for its processing.

Note that a finer load variation resolution can be programmed. However, this possibility added high complexity at computing and managing level, while a very low increase of accuracy in the panel modeling was obtained and was therefore withdrawn.

### 2.2. Control and Acquisition System

The core of this block is a 16 bits MSP430FR5739 microcontroller from Texas Instruments (Dallas, TX, USA). It selects at each moment the resistor to be connected from the load array to the PV module and acquires the main variables involved in the photovoltaic panel characterization: voltages in the panel output, temperature and illuminance. The microcontroller clock rate is set to 24 MHz, letting assume that both temperature and illuminance keep constant while sweeping the 24 resistors in the load module, thus simplifying the measurement process. In addition, this microcontroller includes 10-bit analog-to-digital (ADC) converters.

Environmental temperature is measured by means of a TMP112 digital sensor (Texas Instruments, Dallas, TX, USA). Its measurement range spans from −40 °C to +125 °C, which covers the temperature modelling range (−10 °C to +70 °C) at a suitable accuracy (from 0 °C to 65°C the sensor accuracy is ±0.5 °C, so sensor readings keep this accuracy in almost the full working range, only giving some additional (acceptable) error at the upper and lower limits) and its bias voltage is compatible with the microcontroller requirements.

Considering the measurement of incident solar light, systems designed for industrial solar panel characterization monitor irradiance using pyranometers. Irradiance is a physical magnitude that measures incident solar power per unit area and considers all incident wavelengths. However, pyranometers are bulky and expensive instruments, what make them unsuitable for compact and low-cost systems. Hence, the proposed system incorporates a compact and low-cost MAX44009 illuminance sensor from Maxim Integrated (San Jose, CA, USA). Unlike irradiance, illuminance is a photometric parameter which depends on the human eye sensitivity to colors and has no proportional relation to irradiance. In local measurements, light spectral composition can be assumed as constant during the most of the daylight duration and the relation between photometric and ratiometric magnitudes can be considered fixed [14]. Therefore, if the energy harvesting system is calibrated for a specific location, illuminance offers a suitable measurement of the relative incident solar power for that place. The proposed illuminance sensor presents an input range from 0.045 lux to 188,000 lux, widely covering the illuminance for an average sunny day. In addition, bias voltages are compatible with those of the microcontroller. Data are provided to the microcontroller in a 12-bits floating point representation via I2C protocol.

### 2.3. System Software

**Figure 3:**
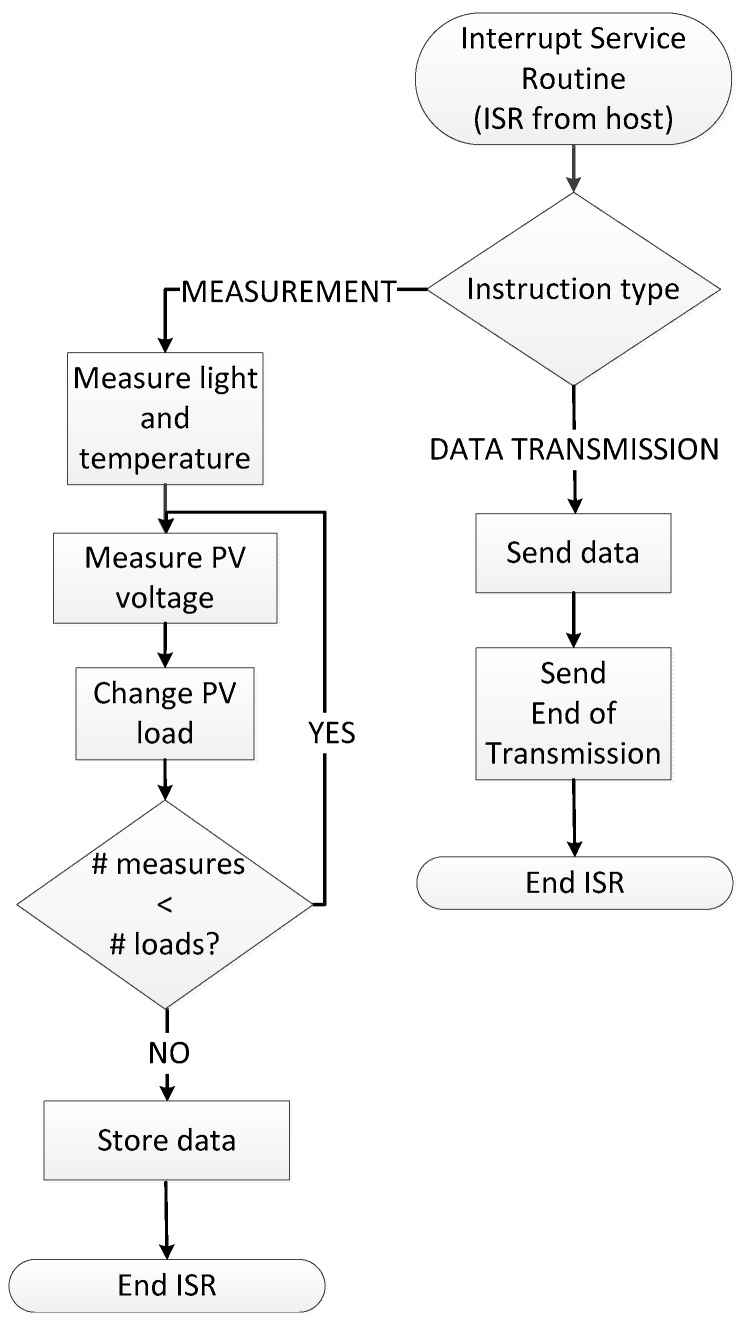
System software dataflow.

The control and acquisition system is designed to be controlled by an external host. Before its deployment, a first characterization can be performed using a computer as host system, though after placement in its definitive location the system is designed to be managed by the sensor node where it is connected. Figure 3 shows the dataflow implemented in the microcontroller. First, the microcontroller configures its peripherals and initializes variables, next going to a low-power state until the host device sends an interrupt request through the serial port. The first interrupt commands the microcontroller to run the measurement process, which starts acquiring temperature and illuminance. Next, the panel output voltage measurements are taken, consecutively selecting the 24 resistors in the variable load. Considering switching, voltage settling time and ADC conversion time using the microcontroller at a 24 MHz working frequency this process takes less than 20 ms, so that both temperature and illuminance can be considered constant along the measurement process.

**Figure 4:**
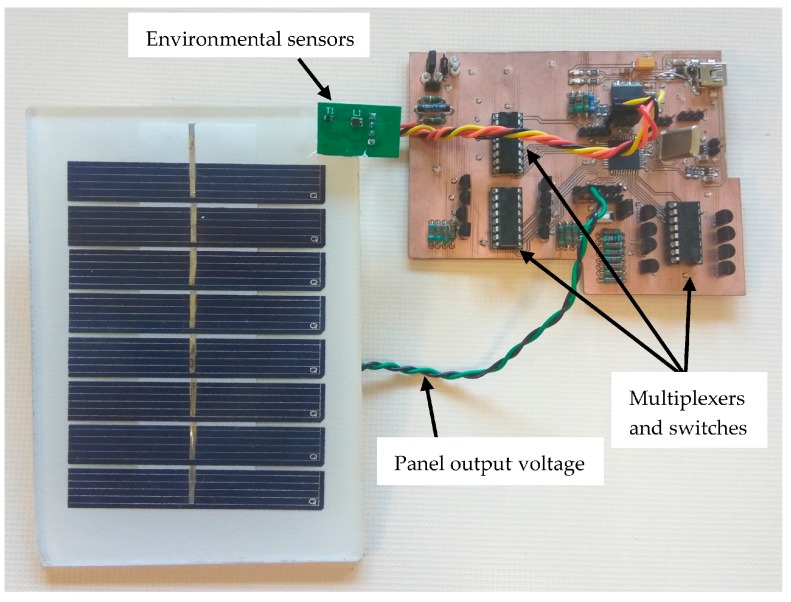
Characterization system connected to the solar panel (control host not shown).

**Figure 5:**
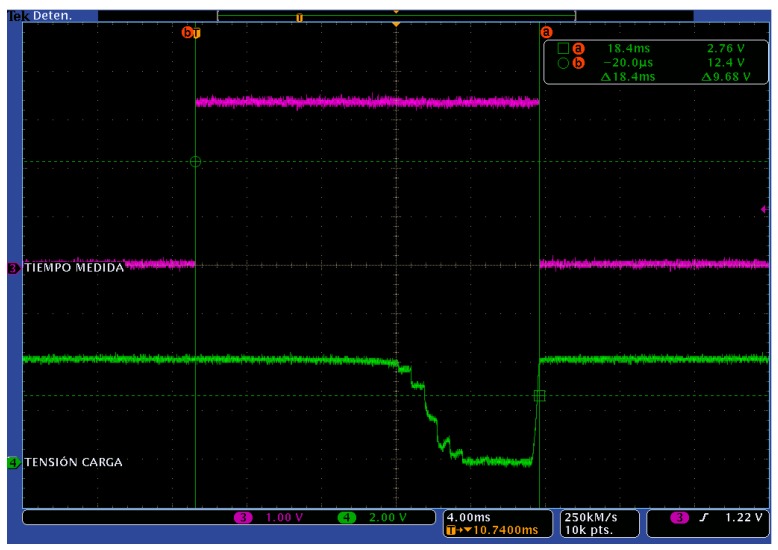
Panel output voltage values (green) in the characterization process sweeping resistor values in the PV panel load. Rising and descent slope in purple signal indicates the start and end time in the measurement. Time span is below 20 ms (see cursor measures).

Figure 4 shows a photograph of the characterization board connected to the solar panel. Illuminance and temperature readings are acquired from the corresponding sensors located in the upper-right side of the PV panel. The power transmitted by the PV panel is estimated from the output voltage readings over the 24 selectable resistors in the variable load (which are distributed in the PCB, see Figure 4). Figure 5 shows the output voltage changes in the solar panel due to variations in the connected load value (green channel). The PV output voltage changes with the resistor load as shown in this figure, where the non-linear region corresponds to resistor values in the range of few kilo-ohms to ohms, in the local irradiance and temperature conditions where the system was tested, being this region directly related to the maximum power point area of interest. The full measurement time is given as the time between cursors a and b in Figure 5, shown as the high level in the upper magenta signal. Though the first 12 ms the measured voltage (green channel) seems to be constant, this is an effect due to the oscilloscope scales; the values measured by the characterization system are different and provide valid data for the panel modelling. The non-monotonicity visible as two glitches in the non-linear region of the load signal appears random and occasionally. These errors can be avoided including a small additional delay between each measurement. However, because these measurements are collected to be used as training data for an artificial neural network to model the PV panel, and considering the robustness of these modelling techniques to local data errors, in order to ensure that environmental parameters (light and temperature) are constant we have preferred keep those times as low as possible. Additionally, this measurement times allows using 0.5 W power dissipation resistors, below the maximum power that the panel can provide (0.8 W) without integrity damage risk, reducing cost and size.

After acquisition, data are locally stored waiting for a transmission command from the host. If a new measurement instruction is received from the host, previous data are overwritten.

### 2.4. Host Control

The panel characterization system operation is designed to be controlled by considering as host device the sensor node where the panel is connected after its deployment in the area to be monitored. In this manner, the periodic PV panel characterization can be included as an additional task in the sensor node operation, thus achieving an updated and accurate local model of the solar panel, with an in situ periodical model recalibration using real temperature, illuminance and transferred power. The re-characterization period can be set according to the user requirements, programming its periodicity from the WSN host by means of the corresponding command. In addition, defining warning conditions a non-periodical characterization can be triggered either by the sensor node itself or by the network host.

The host operation for the panel data acquisition process is the following: after configuration of the serial port that connects to the characterization system, the host sends a command to start the measurement process and waits for a time *t* to the end of this operation. In this time (stated 1 s), the characterization system performs the measurements of temperature, illuminance and power transferred to the resistors in the load array as indicated in Figure 3. Next, the host device sends a data transmission command to the control and acquisition system, receiving the data. This process is recurrently executed with a period of T s, which can be modified according to the requirements, for a sufficiently long time to ensure that measures are available with adequate variability in temperature and illuminance to construct a suitable panel model.

## 3. ANN-Based Photovoltaic Panel Modelling

Using the data collected by means of the proposed characterization system, a numerical model of the solar panel used in this work has been developed. The behavioral model obtained is locally valid under the ambient conditions in which measurements have been taken, and can be updated by collecting new measurements with the characterization system previously presented. This choice simplifies the model, allowing its implementation in compact low-power systems where a microcontroller is the computing core.

The PV panel model has been developed using an artificial neural network (ANN) as fitting technique. ANNs are suitable tools for system modelling in tasks where enough data are available, and no accurate relationship between input and output magnitudes in the modelled system is known. In fact, ANNs applied to power solar panel modelling have been recently reported in the literature [15,16,17].

Among the variety of ANN models, the multilayer perceptron (MLP) is the most common in system modelling due to its universal function estimator characteristic in non-linear fitting tasks, thanks to the use of sigmoid (nonlinear) output functions in its processing nodes [18], and the use of advanced parameter adjustment techniques, as the Levenberg-Marquardt algorithm [19].

Data used to perform the PV model were collected for a week. The ANN input and output data were normalized in the range [−1, +1], considering that environmental inputs (temperature and illumination) as well as output voltage are limited to those shown in Table 2, corresponding to the environmental limits in the region where the panel is working, and the useful panel voltage range. Measures have been acquired every minute, i.e., with a period T = 60 s.

**Figure 6:**
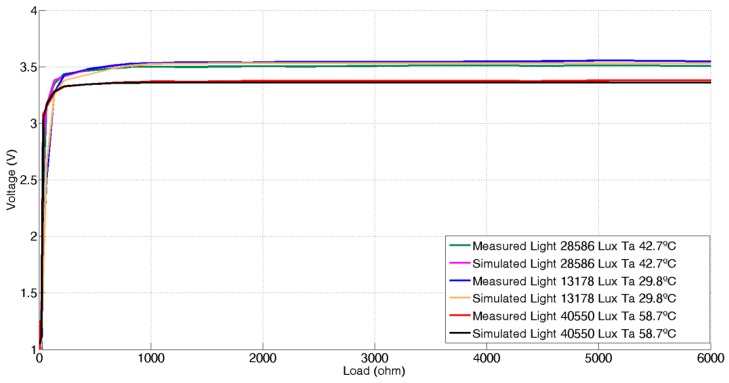
Real and simulated voltage-to-load curves at three different light and temperature conditions.

Figure 6 shows the experimental panel output voltage as a function of the load resistor for three different environmental conditions of illuminance and temperature and its corresponding simulated values provided by a Levenberg-Marquardt trained 3 input – 6 hidden – 1 output MLP, giving very similar results.

**Figure 7:**
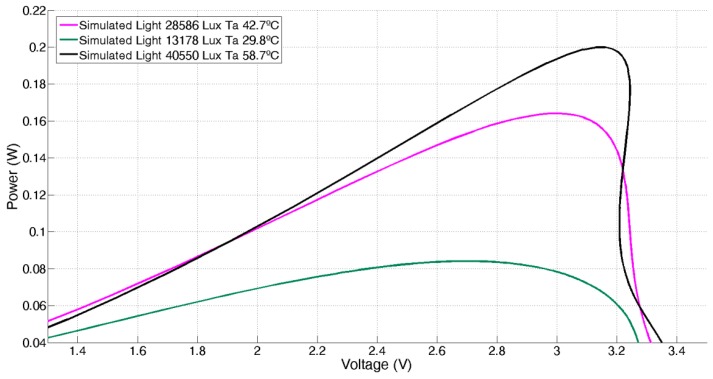
P-V curves obtained from the ANN-based solar panel model.

Once verified the correct fitting, the MLP has been tested to obtain the power-voltage curves that characterize the behaviour of a solar panel. For this, the environmental MLP model inputs (illuminance and temperature) are fixed at the desired values, sweeping the range of the ANN input that represents the panel load to obtain the voltage values from the ANN output. Power is calculated as the output voltage over the corresponding input load value. Figure 7 shows the obtained results. Note that in this modelling, the voltage shows a coupled dependence with illuminance and temperature. This is because, unlike the models developed in laboratory, where temperature and irradiance can be independently controlled, the experimental models based on in-situ environmental measurements account for coupling in temperature and illuminance (i.e., the more illuminance, the more temperature). Therefore, these simpler models are not general, but locally valid and suitable for its use in the proposed application: the development of a solar energy harvesting system based on a local behavioural model to control the maximum power point tracking.

## 4. Solar Energy Harvesting System Architecture

**Figure 8:**
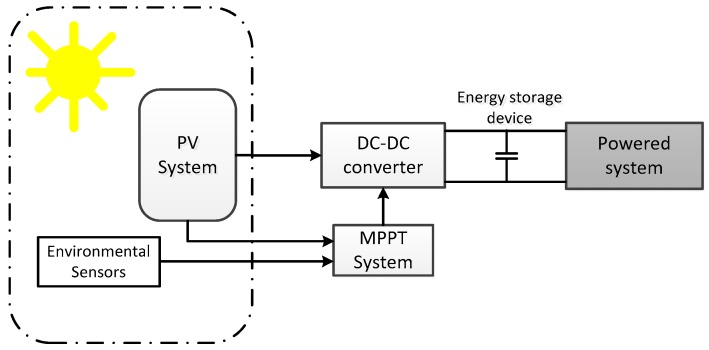
Generic solar energy harvesting block diagram.

Figure 8 shows the block diagram of a typical solar-based energy harvesting system. A photovoltaic device converts solar to electrical energy. The direct-current-to-direct-current (DC-DC) converter generates from the PV device a stabilized voltage, that feds the powered system, comprising an energy storage system (battery, supercapacitor), and the electronic system itself. A maximum power transfer circuit (called maximum power point tracking system, MPPT) tracks environmental and electrical magnitudes from the PV system, and modifies in real time the DC-DC architecture to keep an optimum energy transfer. The monitored variables are selected according to the implemented MPPT technique.

The DC-DC topology depends on the input and output voltages, provided by the solar panel and required by the powered system, respectively. The solar panel provides a maximum voltage of 3.85 V. The energy storage device selected is a supercap, due to its simple control electronic requirements; conversely, batteries require complex control electronics to avoid overload and deep discharges that can damage the load device, as well as higher loading currents, that demand higher panels, unsuitable for the small form factor designs used in wireless CPS. The selected supercap, as will be detailed next, has a maximum input voltage of 5.5 V, suitable for the powered environmental nodes [20]. Under these conditions, the DC-DC architecture has to be a boost converter.

**Figure 9:**
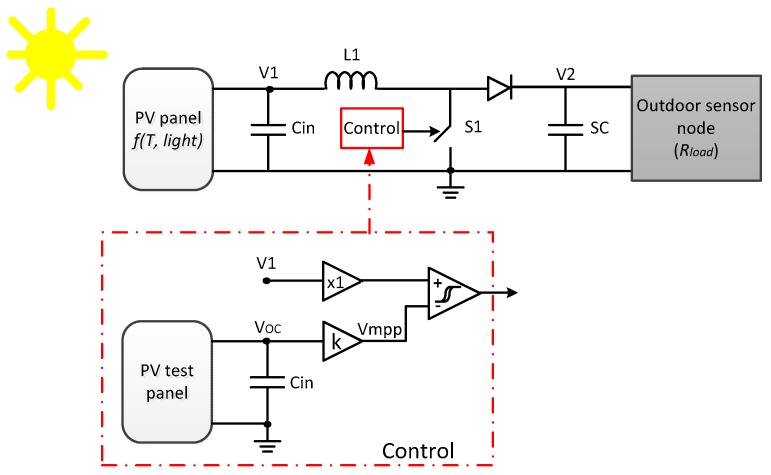
Boost DC-DC circuit. In red, a dual solar panel MPPT control block diagram.

Figure 9 shows the boost DC-DC converter architecture. When switch S1 is ON, the diode is in reverse mode, thus the sensor node (*R_load_*) is powered by the energy stored in the supercap SC, while the inductor L1 is energized from the PV output. When switch S1 is OFF, the output voltage V1 from the PV panel and the energy stored in the inductor load up the supercap voltage (V2), which provides energy directly to the load. Capacitor *C_in_* filters abrupt changes in the output voltage V1 due to variations in the working conditions of PV panel. The component values (Table 3) were estimated using classical techniques [21,22], assuming the system works in continuous conduction mode (CCM) [23].

The value of the supercap must be selected to provide the required energy to the electronic system (the sensor node) for a minimum operating life, even in the absence of light. In our case, the selected device is a 1.5 F–5.5 V EECF5R5U155 supercap from Panasonic (Osaka, Japan), with electrical characteristics compatible with the low dropout (LDO) regulator used in the wireless nodes presented in [20]. The 1.5 F capacitance value guarantee a reduced node lifetime (about 12 h for the aforementioned node), but has been selected to allow a fast and complete characterization of the proposed energy harvesting system. The energy storage capacity can be increased just increasing the capacitance value.

### 4.1. Maximum Power Point Tracking

The maximum power transfer is given at impedance matching, i.e., when the DC-DC regulator presents an input impedance equal to the output impedance of the solar panel. Because the output impedance in the solar panel dynamically depends on its intrinsic characteristics and environmental conditions, impedance fitting requires a dynamic technique able to adapt the DC-DC topology. For this, energy harvesting systems make use of maximum power point transfer (MPPT) algorithms [24], which monitor several different electrical and environmental parameters to come to maximize the power transfer at all times.

The Voltage Open Circuit (VOC) is a widely used MPPT algorithm that tracks the voltage at which the power transfer is maximized. Experimentally, it is known that this voltage is proportional to the system at open circuit, according to:

(1) $$V_{mpp}=k\cdot V_{oc}$$

where *V_mpp_* is the voltage at maximum power point transfer, *V_oc_* is the voltage at open circuit (which depends on the incident light and temperature), and *k* is a constant. The value of *k* depends on the panel model; its value is typically limited to 0.7–0.8 [25,26,27], and can be experimentally estimated by characterizing the solar panel (see Figure 7). In our case, *k* = 0.8.

Therefore, in the VOC MPPT algorithm the tracking of the open circuit voltage is required. For this, there are two different strategies: (1) to add an extra identical solar panel without load to act as a reference or (2) to periodically disconnect the solar panel from the converter and read its voltage at open circuit. Both options present drawbacks: in the first case, an additional cost and increase of system size; in the second, the DC-DC is periodically leaved without power for the time the voltage is measured, losing efficiency.

In the dual panel VOC technique (Figure 9), the control system monitors the open-circuit voltage in the reference PV panel (*V_oc_*), estimating the maximum power transfer voltage *V_mpp_* (Equation (1)), which is compared to the working PV panel voltage *V1*. When *V1* is lower than *V_mpp_*, the switch S1 is turned off, increasing the load impedance, and thus increasing *V1*. Otherwise, if *V1* > *V_mpp_*, the switch is on, reducing the load impedance. To avoid fast and continuous changes in the switch state, the comparator module presents hysteresis.

### 4.2. Maximum Power Point Tracking Technique Based on ANN Modelling

**Figure 10:**
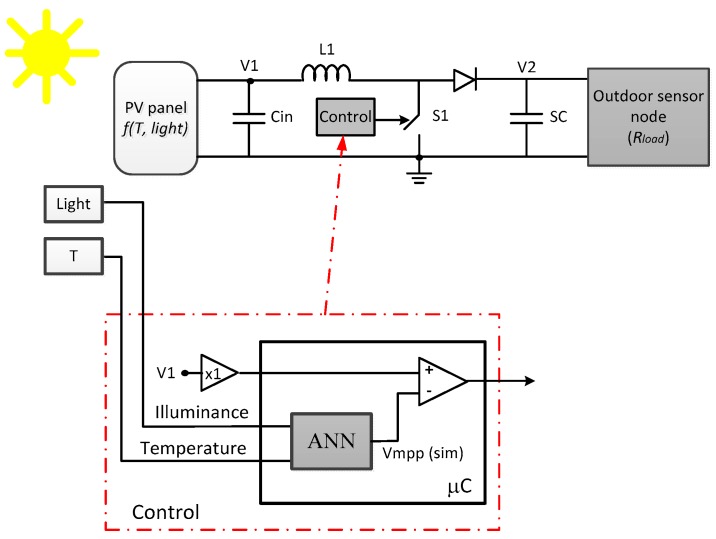
Proposed ANN-based VOC MPPT.

As it was stated before, the use of an additional PV panel to control the switching in the MPPT algorithm presents several drawbacks, mainly cost, size and the need to accurately place both panels in the same orientation to allow right behaviour comparison. In this work, we propose to replace the extra solar panel by the model obtained from an in-situ characterization (Figure 10) achieved using the methodology shown in Section 3. In this way, the proposed tracking method allows continuous energy transfer from the solar panel to the energy storage without the need of additional PV device. This model will be implemented in a low-power microcontroller incorporated to the sensor node where the panel is connected, and it must be capable of simulating the full neural-based model fast enough.

Following similar considerations to that presented in Section 3, the neural model selected is a MLP. In this case, in order to be implemented in a low-cost microcontroller, several constrains next detailed have been applied to the number of input parameters and non-linear function computational complexity, so as to simplify the model while preserving accuracy.

The simulator in the Control block (Figure 10) estimates the *V_mpp_* of the panel from the readings of the sensors of illuminance and temperature; this limits the number of inputs to two. Thus, the PV panel behaviour is modelled by a 2-2-1 MLP with non-linear output function in the hidden layer and linear operation in the output layer. This neural network has been implemented in the same microcontroller used in the panel characterization system (Section 2), a MSP430FR5739 (Texas Instruments, Dallas, TX, USA), which includes a 32 bit hardware multiplier. In this way, the computational resources are shared with the solar panel characterization module. Weight values are represented in 16 bit floating point, and data are normalized in the [−1, +1] range, limiting the training data to the values shown in Table 2. The training data set is generated from the complete ANN panel model previously shown in Section 3, just sweeping the load value for each illuminance-temperature couples (in the valid ranges of the panel location). Therefore, the maximum voltage *Voc* is obtained for each pair of illuminance—temperature values and, hence, the corresponding *V_mpp_* is straightforwardly derived.

**Figure 11:**
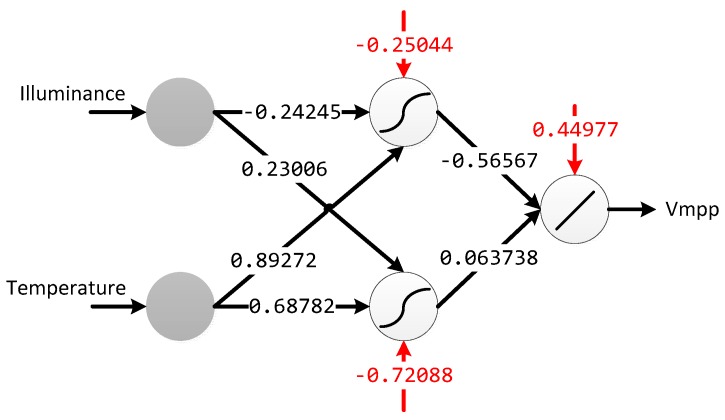
Multilayer perceptron weight values obtained to modelling the solar panel behavior. Red values correspond to bias weights.

The MLP has been trained in off chip mode, using hyperbolic tangent as non-linear operation in the hidden layer, leaving only the execution phase for its electronic implementation. Figure 11 shows the MLP architecture with the resulting weights after training.

Due to its inherent computational complexity, the most important limitation when a neural model is electronically implemented is the non-linear operation in the hidden processors. In this case, the hyperbolic tangent has been replaced by a five sections piecewise polynomial approach given by:

(2) $$y=f\left(x\right)=\{\begin{array}{c} -1,\;x\leq -5.3\\ 0.0127\cdot x^{3}+0.1435\cdot x^{2}+0.5234\cdot x-0.3840,\;-0.9>x>-5.3\\ -0.2358\cdot x^{3}+0.9834\cdot x,\;-0.9\leq x\;\leq 0.9\\ 0.0127\cdot x^{3}-0.1435\cdot x^{2}+0.5234\cdot x+0.3840,\;5.3>x>0.9\\ +1,\;x\geq 5.3 \end{array}$$

**Figure 12:**
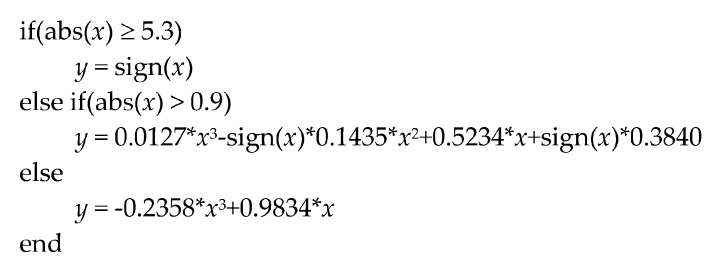
Pseudocode representing the piecewise polynomial implementation of equation.

**Figure 13:**
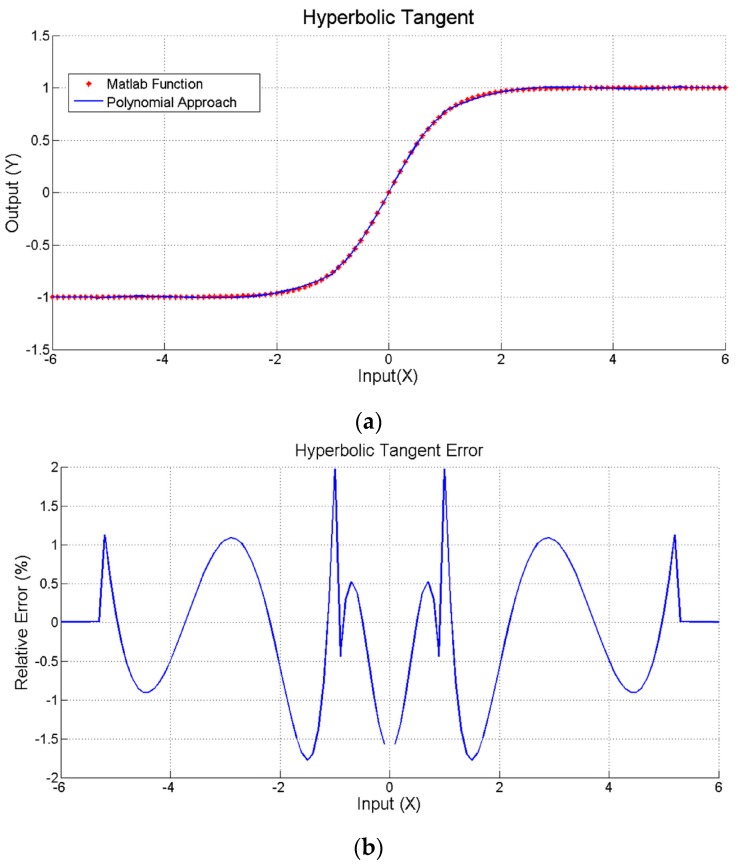
(**a**) Hyperbolic tangent computation using a standard C library *tanh(x)* function (red) and the piecewise approach (blue) on a microcontroller; (**b**) Relative error of the polynomial fitting compared to the library function.

**Figure 14:**
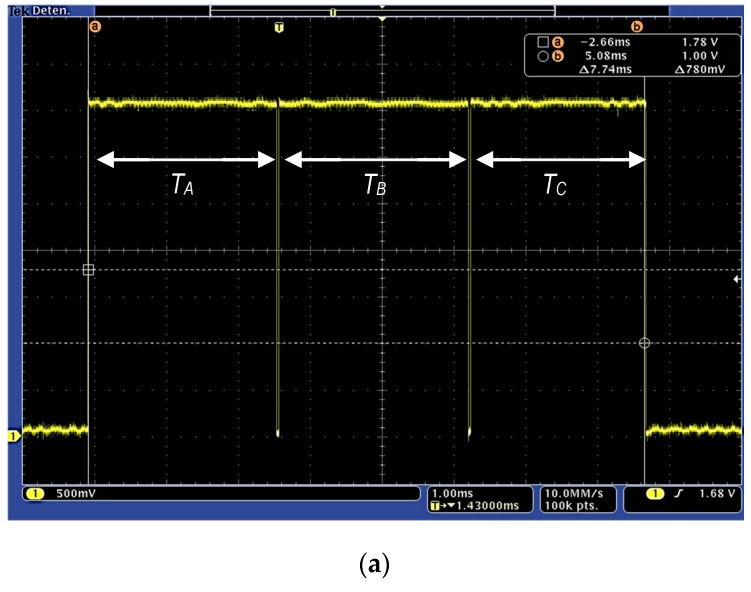

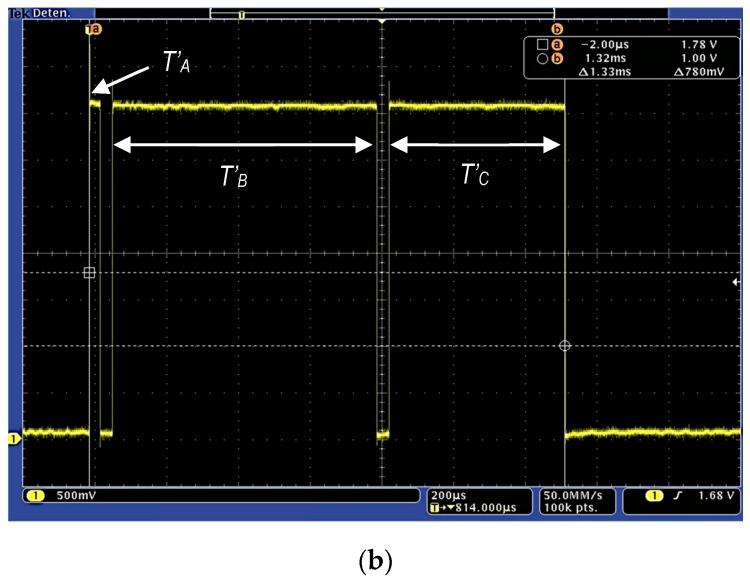
Computing time measurement for three different input values using (**a**) C library-based *tanh(x)* operation and (**b**) polynomial approach; Times correspond, respectively, to calculate the operations for inputs that meet: abs(*x*) ≥ 5.3 ($T_{A}$, $T_{A}^{\prime }$); 5.3 > abs(*x*) > 0.9 ($T_{B},\;T_{B}^{\prime }$); and abs(x) ≤ 0.9 ($T_{C},\;T_{C}^{\prime }$).

And that has been implemented minimizing the operations, as shown in Figure 12, to reduce the computing time. The accuracy of this polynomial approach is shown in Figure 13. Left side shows the representation of a *tanh(x)* computed by Matlab running on a personal computer overlapped to the proposed polynomial approach in Figure 12 implemented on the used microcontroller. Right side in Figure 13 presents the relative error of this approach, which is lower than 2% (except in the zero crossing). Finally, the execution time has been experimentally estimated and compared to the time required for computing the *tanh(x)*, using the standard C mathematical library. Both operations have been implemented in the microcontroller, measuring the execution time switching an output pin from logical ‘1’ to logical ‘0’. Figure 14 shows the operation time using the *tanh(x)* (a) and polynomial approach (b) for three different input values, corresponding to the input ranges shown in Figure 12. The *tanh(x)* function requires a constant time to perform each of the three operations ($T_{A}$ = $T_{B}$ =$T_{C}$ = 2.4 ms), while the polynomial implementation requires a variable time of $T_{C}^{'}\;$= 740 μs (worst case, second *if(.)* in Figure 12), $T_{B}^{\prime }\;$= 500 μs (*else* condition) and $T_{A}^{\prime }\;$= 33 μs (best case, first *if(.)*), always improving the execution time.

**Figure 15:**
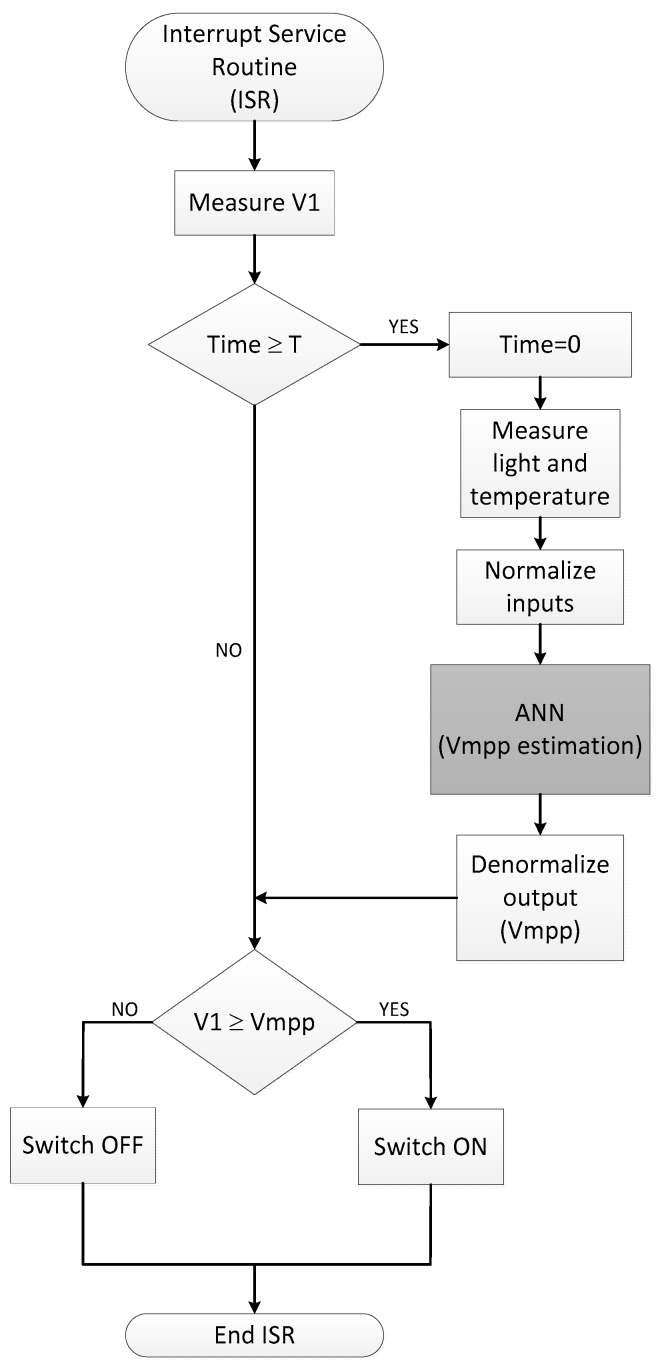
ANN-based VOC MPPT routine flow diagram.

Figure 15 shows the flow diagram of the VOC MPPT routine with ANN modelling of the PV panel. The Control block microcontroller enters every time *t* in the interrupt routine. If the time after the last *V_mpp_* estimation is higher than a set time *T* (>*t*), the system updates its value by computing the new *V_mpp_* from the ANN model using current illuminance and temperature readings. Next, if the voltage V1 at the panel output is higher than the *V_mpp_*, the control switch is turn on reducing the input impedance and, hence, reducing the voltage V1. Otherwise, the switch turns off.

### 4.3. MPPT Comparison

**Figure 16:**
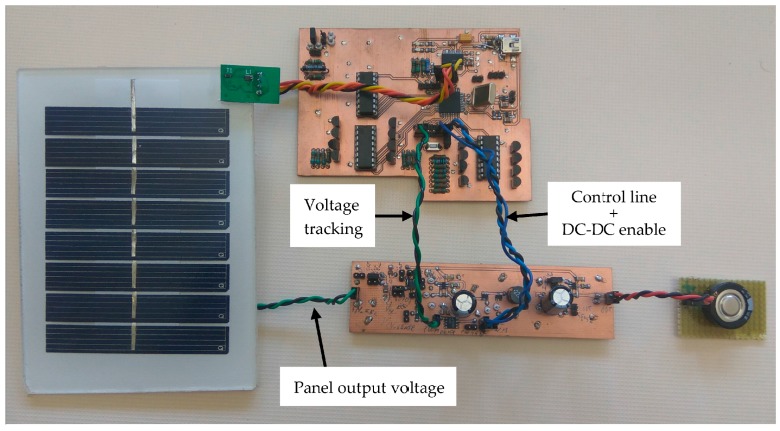
Full solar energy harvesting system based on ANN MPPT and energy storage system (supercapacitor): solar panel (left), control system (centre, on top, includes the characterization system), DC-DC system (centre, bottom) and supercap (right). In this case, only one of the 1.5 F supercaps is connected to the boost system for characterization purposes.

Figure 16 shows the electronic implementation of the energy harvesting system based on the VOC technique with ANN-based panel modelling. Temperature and illuminance are monitored by the microcontroller (located at the characterization system on top of the figure), that compares the real panel output voltage (sent from the DC-DC system to the microcontroller through the voltage tracking wires), and the ANN-based maximum power point voltage estimation (from temperature and illuminance real time values) to properly switch the value of the Control line of the boost DC-DC converter (centre, bottom). An additional line (in parallel to the Control line in Figure 16) disables the DC-DC circuit to operate the characterization system when it is required. Load tests were performed using one supercap to speed up the comparison process, though a wireless sensor node can require several in parallel [20] for suitable energy storage.

The proposed technique has been compared to a classical analog VOC MPPT to evaluate its performance. In the proposed model, the MPPT management routine is executed every 10 μs (*t* in Figure 15), while the *V_mpp_* estimation is upgraded every 100 ms (*T* in Figure 15). Both techniques are executed in parallel over two identical solar panels with the same orientation, seeking for the same working conditions.

**Figure 17:**
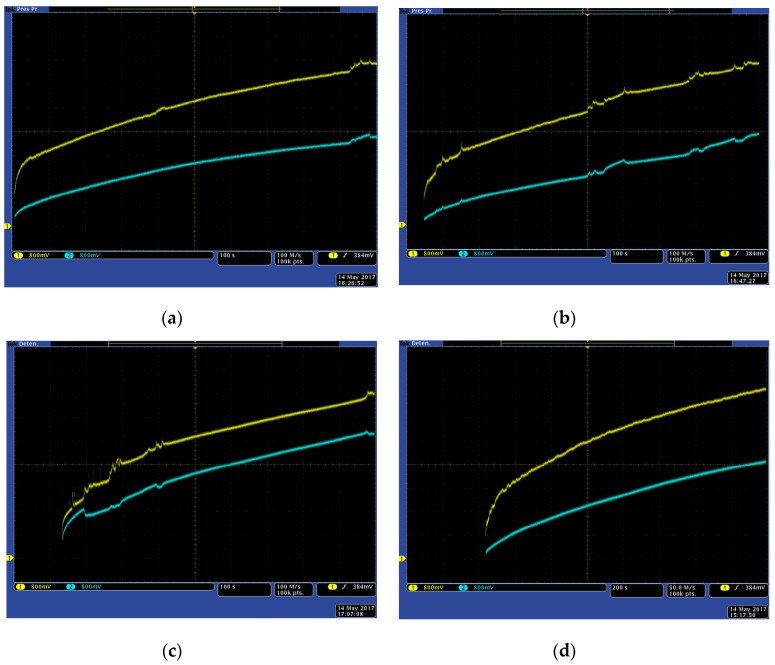
Charge curves for a 1.5 F supercap for a VOC algorithm using a reference panel (blue) and the ANN-based solar panel model (yellow). Environmental conditions (irradiance and temperature, average): (**a**) 850 W/m^2^, 35 °C; (**b**) 1300 W/ m^2^, 55 °C; (**c**) 1120 W/ m^2^, 43 °C; (**d**) 500 W/ m^2^, 26 °C.

Figure 17 shows the dynamic energy load process for a 1.5 F supercap using both classical (blue) and ANN-based (yellow) VOC MPPT techniques at four different environmental conditions. The load process stops when the supercap is fully loaded and its voltage reaches 5.5 volts. In Figure 17, the x axis represents time (1000 seconds full screen for Figure 17a–c, and 2000 seconds for Figure 17d), while y axis shows the voltage evolution with time. Both techniques have been implemented in parallel, placed in identical environmental conditions and orientation. Energy load processes are triggered at the same time, and sensors and DC-DC converters are identical for both systems, being the control architecture the only difference. The proposed system presents a more efficient behaviour under those conditions, considerably reducing the load time.

## 5. Conclusions

The application of a characterization system for small solar panels powering wireless sensor networks after deployment provides an accurate local behavioural model, which allows monitoring the efficiency changes in the energy generation due to both environmental and aging effects. Taking this into account, this paper has proposed an in situ PV panel modelling system, complying with the severe constraints related to wireless sensor networks in size, cost and energy consumption. Based on the acquired data, a behaviour-based PV model is developed based on a multilayer perceptron; this ANN solution makes easier to incorporate new characterization measures into the model, adapting it to changes in the current operating conditions.

Finally, a boost DC-DC converter based on the maximum power point tracking using the VOC algorithm, where the pilot panel monitoring the open circuit voltage is replaced by a customized behavioural model based on the proposed multilayer perceptron approach, with reduced computing requirements to allow the software model to be implemented in a low-cost microcontroller embedded in the wireless node, that manages both the data acquisition and modelling. Compared to the classical VOC technique, experimental results show an improved performance in loading a supercap as energy storage device. In addition, this technique can be applied to different MPPT algorithms where a solar panel or its behaviour is required in its operation.

## Figures and Tables

**Table 1:** Resistor values in the variable load module.

SEL(1:3)	R_MUX1_ (Ω)	R_MUX2_ (Ω)	R_MUX3_ (Ω)
000	10,000	1800	62
001	8200	1200	33
010	7500	1000	16
011	6200	820	8.2
100	5100	680	3.9
101	4300	430	2
110	3300	220	1
111	2200	130	0.1

**Table 2:** Range of parameters used in the solar panel modelling.

Parameter	Upper Limit	Lower Limit
Light (lux)	120,000	10,000
Temperature (°C)	70	−10
Voltage (V)	5	2.8
Panel load (Ω)	10,000	0.1

**Table 3:** Component values for the DC-DC boost converter.

Component	Value
Cin	>25 µF
L1	>6.7 mH
Diode	HSMS-2800
Switch	IRFML8422 (MOSFET transistor)
